# Positive and negative well-being and objectively measured sedentary behaviour in older adults: evidence from three cohorts

**DOI:** 10.1186/s12877-019-1026-1

**Published:** 2019-01-30

**Authors:** Judith A. Okely, Iva Čukić, Richard J. Shaw, Sebastien F. Chastin, Philippa M. Dall, Ian J. Deary, Geoff Der, Manon L. Dontje, Dawn A. Skelton, Catharine R. Gale, on behalf of the Seniors USP team, Dawn A. Skelton, Sebastien Chastin, Simon Cox, Elaine Coulter, Iva Čukić, Philippa Dall, Ian Deary, Geoff Der, Manon Dontje, Claire Fitzsimons, Catharine Gale, Jason Gill, Malcolm Granat, Cindy Gray, Carolyn Greig, Elaine Hindle, Karen Laird, Gillian Mead, Nanette Mutrie, Victoria Palmer, Ratko Radakovic, Naveed Sattar, Richard Shaw, John Starr, Sally Stewart, Sally Wyke

**Affiliations:** 1 Centre for Cognitive Ageing and Cognitive Epidemiology, Department of Psychology, University of Edinburgh, 7 George Square, Edinburgh, EH8 9JZ UK; 2 MRC/CSO Social and Public Health Sciences Unit, University of Glasgow, Glasgow, UK; 3 Institute for Applied Health Research, School of Health and Life Sciences, Glasgow Caledonian University, Glasgow, UK; 4 Department of Movement and Sports Sciences, Faculty of Medicine and Health Science, Ghent University, Ghent, Belgium; 5 School of Population and Global Health, University of Western Australia, Perth, Australia; 6 MRC Lifecourse Epidemiology Unit, University of Southampton, Southampton, UK

**Keywords:** Wellbeing, Depression, Anxiety, Sedentary behaviour, Tri-axial inclinometer

## Abstract

**Background:**

Sedentary behaviour is related to poorer health independently of time spent in moderate to vigorous physical activity. The aim of this study was to investigate whether wellbeing or symptoms of anxiety or depression predict sedentary behaviour in older adults.

**Method:**

Participants were drawn from the Lothian Birth Cohort 1936 (LBC1936) (*n* = 271), and the West of Scotland Twenty-07 1950s (*n* = 309) and 1930s (*n* = 118) cohorts. Sedentary outcomes, sedentary time, and number of sit-to-stand transitions, were measured with a three-dimensional accelerometer (activPAL activity monitor) worn for 7 days. In the Twenty-07 cohorts, symptoms of anxiety and depression were assessed in 2008 and sedentary outcomes were assessed ~ 8 years later in 2015 and 2016. In the LBC1936 cohort, wellbeing and symptoms of anxiety and depression were assessed concurrently with sedentary behaviour in 2015 and 2016. We tested for an association between wellbeing, anxiety or depression and the sedentary outcomes using multivariate regression analysis.

**Results:**

We observed no association between wellbeing or symptoms of anxiety and the sedentary outcomes. Symptoms of depression were positively associated with sedentary time in the LBC1936 and Twenty-07 1950s cohort, and negatively associated with number of sit-to-stand transitions in the LBC1936. Meta-analytic estimates of the association between depressive symptoms and sedentary time or number of sit-to-stand transitions, adjusted for age, sex, BMI, long-standing illness, and education, were *β* = 0.11 (95% CI = 0.03, 0.18) and *β* = − 0.11 (95% CI = − 0.19, −0.03) respectively.

**Conclusion:**

Our findings indicate that depressive symptoms are positively associated with sedentary behavior. Future studies should investigate the causal direction of this association.

## Background

Sedentary behaviour, defined as any waking behaviour in a seated or reclined posture that involves an energy expenditure of ≤1.5 metabolic equivalent of task [1], is related to poorer health independently of time spent in moderate to vigorous activity [2–8]. This finding has informed public health guidelines: in addition to recommending engagement in moderate physical activity, the UK Department of Health now advises that adults over the age of 65 should minimise the time they spend being sedentary for extended periods [9]. However, levels of sedentary behaviour remain high, particularly among older adults, with those aged 60 or over spending an average of 9 h sedentary per day [10]. Identification of modifiable determinants of sedentary behaviour in older age would help inform the development of behaviour change interventions. Here, we examine the potential effect of three psychosocial factors, subjective wellbeing and symptoms of anxiety or depression, on patterns of sedentary behaviour.

Subjective wellbeing, anxiety and depressive symptoms predict a range of health behaviours including physical activity. Individuals who report high wellbeing tend to follow a healthier lifestyle [11–14], whereas those who experience symptoms of anxiety or depression are less likely to engage in health protective practices [15–17]. There is evidence that these associations are bi-directional. For instance, treatment of clinical depression is typically followed by an increase in physical activity [18] whereas physical activity intervention studies show that physical activity can alleviate depressive symptoms and positively impact wellbeing [19, 20]. In this study, we focus on the potential impact of wellbeing, anxiety and depression on sedentary behaviour. We chose to include both positive and negative wellbeing measures as findings from previous studies indicate that these measures can be differentially related to health behaviours [21–23].

The potential impact of positive and negative wellbeing on sedentary behaviour was recently identified as a research priority by the European Joint Programme Initiative for action on diet, physical activity and health (DEDIPAC) [24]. However, knowledge regarding the association between these psychosocial factors and sedentary behaviour in older adults remains limited [25]. Several cross-sectional studies found a positive association between symptoms of anxiety or depression and sedentary behaviour in adult or student populations [26–31]. However, only four studies examined these associations in older adults. In a cross-sectional study of 1580 Japanese older adults, psychological distress was positively associated with some (self-reported) sedentary activities (watching television, or sitting around) but not others (computer use or reading). Similar results are reported by a study of 6359 English older adults; self-reported TV viewing time was associated with more depressive symptoms whereas internet use was associated with fewer depressive symptoms [32]. By contrast, two cross-sectional studies of older adults, found no association between time spent watching television or a hip-worn accelerometer derived measure of sedentary behaviour and symptoms of anxiety or depression [33, 34].

The construct of wellbeing consists of three subdomains: life satisfaction, life meaning or purpose, and the experience of positive affect [35]. There is some evidence that positive affect is negatively associated with sedentary behaviour. Elavsky, Kishida, and Mogle [36] examined concurrent and lagged associations between momentary positive affect (assessed four times a day) and sedentary behaviour (measured continuously by hip-worn accelerometer) in a sample of 121 middle aged women over 15 days. The authors found an inverse association between concurrent positive affect and sedentary behaviour; however, positive affect did not predict subsequent levels of sedentary behaviour. Two studies tested for an association between wellbeing and sedentary behaviour in older adults. In a cross-sectional study involving 228 older adults, Withall et al. [37] found no association between wellbeing and a hip-worn accelerometer derived measure of sedentary behaviour. In a sample of 100 older adults, Maher and Conroy [38] found that sedentary behaviour, assessed with thigh-worn activity monitors, was associated with life satisfaction at the within person level, such that participants were less sedentary on days when they reported higher life-satisfaction. However, between participant differences in mean life satisfaction were not related to between participant differences in sedentary behaviour [38].

An important limitation of studies into the psychosocial correlates of sedentariness in old age relates to the measurement of sedentary behaviour. Previous studies employed either self-report or accelerometer derived measures. Self-reported sedentary behaviour may be unreliable due to a combination of underestimation [10, 39–41] and low precision [42]. Therefore, studies that employed this method [29, 32, 33] may have underestimated associations between psychosocial factors and sedentary behaviour. Other studies measured sedentary behaviour using hip-worn accelerometers [34, 37, 38]. However, these measures can also be unreliable as hip-worn accelerometers do not record postural changes, e.g., transitions from sitting to standing [37]. Tri-axial inclinometers such as activPal3 (PAL Technologies Ltd., Glasgow, UK), can detect postural changes and thus provide a valid measure of sedentary behaviour [43]. Only one previous study [38] used thigh-worn accelerometers measuring postural sitting. However, this latter study treated sedentary behaviour as a predictor of life satisfaction rather than vice versa. A further limitation was that it did not test for an association between negative psychosocial factors and sedentary behaviour.

In the present study, we used three-dimensional accelerometers (PAL Technologies Ltd., Glasgow, UK) to measure sedentary behaviour. This approach allowed us to address previous limitations, described above, relating to the reliability of sedentary behaviour measurement. In addition to total time spent sedentary, these accelerometers record frequency of sit-to-stand transitions. Previous observational studies have shown that breaking up sedentary time with sit-to-stand transitions is associated with a reduction in metabolic risk factors including obesity, elevated blood glucose, and triglyceride levels, and, that these associations are partially independently of total sedentary time and physical activity [43–49]. These findings have prompted the suggestion that sit-to-stand transitions could provide a target for interventions to reduce the health consequences of sedentariness [50]. This approach could be particularly applicable among older people who are unable to engage in more vigorous forms of exercise [50]. Research into the determinants of sit-to-stand transitions is at an early stage, and the psychosocial predictors of this behaviour are not yet known.

In the present study, we used activPAL3 activity monitors (PAL Technologies Ltd., Glasgow, UK) to measure sedentary behaviour in three cohorts of older adults. Our aim was to test whether wellbeing or symptoms of anxiety or depression assessed either concurrently or 8 years previously predict objectively measured sedentary time or number of sit-to-stand transitions.

## Methods

### Participants

We used data from the Seniors USP (Understanding Sedentary Patterns) study. Participants in the Seniors USP study were recruited from three cohorts of older adults: the Lothian Birth Cohort 1936 (LBC1936), the West of Scotland Twenty-07 1950s cohort (Twenty-07 1950s) and the West of Scotland Twenty-07 1930s cohort (Twenty-07 1930s). Ethical approval was obtained from the Multi-Centre Research Ethics Committee for Scotland (LBC1936). Ethical approval was obtained from the NHS and/or Glasgow University Ethics Committees (Twenty-07 cohorts). All participants provided written informed consent.

#### LBC1936

The LBC1936 is a follow up study of the Scottish Mental Survey 1947. Participants, all born in 1936, were recruited from the Lothian area of Scotland. See Deary, Gow, Pattie, and Starr [51] and Deary et al. [52] for details regarding recruitment and testing procedures.

Participants were recruited for the Seniors USP study during wave 4 of LBC1936 (in 2015 and 2016). The target number of LBC1936 participants for the Seniors USP study was 300. Of the 373 participants who were approached, 304 agreed to take part in the Seniors USP study and had an activity monitor fitted. Of these 304 participants, 302 returned the monitor.

#### Twenty-07 study

The Twenty-07 study consists of three cohorts: the Twenty-07 1930s, 1950s, and 1970s cohorts. Participants, born in 1930s, 1950s, and 1970s, were recruited from the Central Clydeside area of Scotland in 1987. The main Twenty-07 study ended in 2008, following 5 waves of data collection. See Benzeval et al. [53] for further details regarding recruitment and assessment procedures.

Seniors USP participants were recruited from the Twenty-07 1930s and 1950s cohorts in 2015 and 2016. All Twenty-07 1930s and 1950s cohort participants living in the greater Glasgow area were eligible to take part. All eligible participants in the 1930s cohort (*n* = 468) were approached, of whom 129 agreed to wear the activity monitor. A random sample of eligible people (*n* = 765) in the 1950s cohort were approached, of whom 340 agreed to take part.

#### Analytical samples

Participants were excluded if they had missing sedentary or covariate data. Analytical samples sizes were 271, 118, and 309, for the LBC1936, Twenty-07 1930s, and the Twenty-07 1950s cohorts, respectively. Additionally, five LBC1936 participants with missing Warwick–Edinburgh Mental Wellbeing Scale (WEMWBS) data were excluded in analysis with the wellbeing scale, and one LBC1936 participant with missing Hospital Anxiety and Depression Scale (HADS) data was excluded from analysis with the HADS depression and anxiety subscales. Finally, 13 participants were excluded from the Twenty-07 1950s cohort and six from the Twenty-07 1930s cohort for analysis with the HADS anxiety and depression subscales due to missing data on these variables.

### Measures

#### Sedentary behaviour

Sedentary behaviour was measured using an activPAL activity monitor (activPAL3c, PAL Technologies Ltd., Glasgow, UK) which is a tri-axial accelerometer that continuously monitors the inclination of the thigh. This measure is well validated and reliable [43, 54, 55]. The activPAL monitor, was fitted to the front of the thigh on the dominant leg using a waterproof dressing. Participants were asked to wear the monitor continuously for a minimum of 7 days in order to provide a seven-day continuous recording (7 × 24 h starting at midnight) of activity. While they wore the monitor, participants were asked to record in a daily diary the time at which they got into and out of bed, and to estimate the time at which they fell asleep and woke up.

Recorded activPal data were downloaded using activPAL software version 7.2.32 (PAL Technologies Ltd., UK), and collated with sleep diary data using the statistical package R (R Core Team, 2016). We then created two outcome measures: the average percentage of waking time spent sedentary per day (hereafter sedentary time), and the average number of sit-to-stand transitions per day (hereafter number of sit-to-stand transitions). The protocol for obtaining the outcome measures was consistent across all three cohorts in this study. Only those participants with 7 days of activity data were included in analyses.

#### Symptoms of anxiety and depression

The Hospital Anxiety and Depression Scale (HADS) [56] was used to measure symptoms of anxiety and depression in all three cohorts. The scale can be divided into anxiety and depression subscales, each consisting of 7 items. Higher scores indicate more symptoms of anxiety or depression; the highest possible score for each subscale is 21 [56]. The HADS has been validated in general and elderly populations [57].

#### Wellbeing

Wellbeing was measured with the Warwick–Edinburgh Mental Wellbeing Scale (WEMWBS). This measure was available for the LBC1936 cohort but not the Twenty-07 cohorts. The WEMWBS is a 14 item scale designed to measure positive mental health. Possible scores range from 14 to 70 with higher scores indicating higher wellbeing. The WEMWBS has been validated in a representative general population sample of British adults [58].

#### Covariates

We treated age, sex, body mass index (BMI), educational attainment and history of limiting long-standing illness or disability as covariates. BMI was calculated as weight (in kilograms) divided by height squared (in meters). Trained research nurses measured participants’ height and weight. Educational attainment was coded as either no qualification, basic qualification (including O levels and A levels), or advanced qualification (including semi-professional and professional occupations, or a degree). Participants reported whether they had a long-standing illness, disability or infirmity and, whether the condition limited their activities in any way. Participants were coded as having a limiting illness or disability only if the condition limited their activities. Covariates were consistent across all three cohorts.

#### Measure timing

For the LBC1936 cohort, all measures, apart from educational attainment, were from wave 4 of the study (2015/6). Data regarding educational attainment was taken from wave 1 (2004–2007). For the Twenty-07 cohorts, sedentary behaviour, age, weight (to calculate BMI), educational attainment and limiting illness or disability were assessed in 2015/6; however, HADS and height (to calculate BMI) measures were taken from the final wave of the Twenty-07 study in 2008. The timing of these assessments is also detailed in Table 1.

**Table 1:** Descriptive statistics of the three cohorts

	LBC1936	Twenty-07 1930s	Twenty-07 1950s
	*n* = 271	*n = 118*	*n* = 309
Age *M (SD)*	78.97 (0.44)	83.40 (0.62)	64.58 (0.90)
Sedentary time (%) *M (SD)*	62.51 (10.38)	68.16 (10.93)	60.84 (10.77)
Sit-to-stand (number) *M (SD)*	43.97 (11.49)	42.85 (13.60)	49.12 (13.63)
WEMWBS *M (SD)*	51.81 (7.93)	N/A	N/A
HADS-A *Mdn* (IQR)	4 (2–6)	5 (3–7)^a^	6 (4–8)^a^
HADS-D *Mdn* (IQR)	2 (1–4)	2 (1–5)^a^	2 (1–5)^a^
BMI *M* *(SD)*	27.22 (4.30)	27.72 (4.57)^b^	27.92 (4.55)^b^
Education *n (%)*			
Low	36 (13.3)	34 (28.8)	25 (8.1)
Medium	133 (49.1)	61 (50.8)	161 (51.9)
High	102 (37.6)	24 (20.3)	124 (40.0)
Limiting illness/disability *n (%)*	49 (18.1)	50 (42.4)	60 (19.4)

*M* mean, *SD* standard deviation, *Mdn* median, *IQR* interquartile range, *BMI* body mass index, *WEMWBS* Warwick–Edinburgh Mental Wellbeing Scale, *HADS-A/D* Hospital Anxiety and Depression Scale-Anxiety/Depression subscale

^a^ HADS measured approximately 8 years before sedentary behaviour assessment

^b^ Height measured approximately 8 years before weight and sedentary behaviour assessment

### Statistical analysis

Analyses were performed in RStudio 3.4.1 [59]. We ran linear regression models to test for an association between wellbeing, symptoms of anxiety or depression and the sedentary behaviour measures. Sedentary time, and sit-to-stand transitions were treated as dependent variables. The sit-to-stand transitions variable was not normally distributed, we therefore used a square root transformed version of this variable in our analysis. We tested for an association between each of the psychosocial factors (wellbeing, anxiety or depression) and the outcomes of sedentary time or sit-to-stand transitions in turn, this approach resulted in 6 separate models (3 psychosocial predictors × 2 sedentary behaviour outcomes). Each model was adjusted for age and sex followed by additional adjustment for BMI, educational attainment, and limiting illness or disability. *P*-values were corrected for multiple comparisons using the false discovery rate (FDR) approach [60]. This correction was carried out for the number of tests within each cohort separately.

The HADS depression subscale includes an item related to sedentary behaviour: “I feel as if I am slowed down”. To test whether any associations between depression and sedentary behaviour were driven by this item, we created a modified HADS depression score excluding responses to this item. We then we re-ran regression analysis where the HADS depression subscale was entered as a predictor, replacing the original HADS depression scale with the modified version.

With sample sizes ranging from 118 to 309, we had 80% power to detect effect sizes ranging from 0.25 to 0.16. However, the effect size of the association between psychosocial measures and sedentary outcomes could be smaller. Symptoms of anxiety and depression were assessed using identical methods in all three cohorts. Thus, in order to increase power, we meta-analysed estimates of the association between the HADS subscales (anxiety or depression) and the outcomes of sedentary time or sit-to-stand transitions.

## Results

### Descriptive statistics

Table 1 provides descriptive statistics of all the variables in the study. Of the three cohorts, participants in the Twenty-07 1930s cohort (mean age 83) were the most sedentary, spending approximately 68% of their daily waking time sedentary. The LBC1936 cohort (mean age 79) spent approximately 63% of their daily waking time sedentary. Participants in the Twenty-07 1950s cohort (mean age 65) spent 61% of their daily waking time sedentary – making them the least sedentary group.

Median depression scores were similar in all three cohorts. Median anxiety scores were highest in the Twenty-07 1950s cohort (Median [*Mdn*] = 6, Interquartile range [IQR] = 4–8), followed by the Twenty-07 1930s cohort (*Mdn =* 5, IQR = 3–7), and were lowest in the LBC1936 cohort (*Mdn* = 4, IQR = 2–6).

### Predicting sedentary behaviour

Table 2 displays standardised betas and 95% confidence intervals (95% CI) from the regression analysis. *P*-values were corrected for multiple comparisons [60].

**Table 2:** Standardised betas (95% CIs) for the association between wellbeing or symptoms of anxiety or depression and average daily sedentary time or number of sit-to-stand transitions

	Sedentary time		Sit-to-stand transitions	
	*β* (95% CI)	*p*	*β* (95% CI)	*p*
LBC1936				
WEMWBS				
+ age, sex	−0.04 (− 0.16, 0.08)	.555	0.09 (− 0.03, 0.21)	.258
+ multiv. Adj.	−0.05 (− 0.17, 0.07)	.407	0.12 (0.00, 0.24)	.136
HADS-A				
+ age, sex	−0.08 (− 0.19, 0.03)	.258	0.08 (− 0.04, 0.20)	.258
+ multiv. Adj.	−0.05 (− 0.06, 0.18)	.407	0.08 (− 0.04, 0.20)	.287
HADS-D				
+ age, sex	**0.14 (0.02, 0.26)**	.045	**−0.17 (− 0.29, − 0.05)**	.024
+ multiv. Adj.	0.11 (− 0.01, 0.23)	.136	**−0.19 (− 0.31, − 0.07)**	.012
Twenty-07 1950s				
HADS-A				
+ age, sex	0.13 (0.01, 0.27)	.068	−0.03 (− 0.09, 0.15)	.876
+ multiv. Adj.	0.05 (− 0.07, 0.17)	.692	0.04 (− 0.08, 0.17)	.692
HADS-D				
+ age, sex	**0.19 (0.07, 0.30)**	.004	−0.02 (− 0.14, 0.09)	.876
+ multiv. Adj.	0.08 (− 0.03, 0.20)	.644	0.00 (− 0.12, 0.13)	.959
Twenty-07 1930s				
HADS-A				
+ age, sex	−0.07 (− 0.28, 0.12)	.728	0.07 (− 0.12, 0.26)	.624
+ multiv. Adj.	− 0.17 (− 0.37, 0.04)	.151	0.12 (− 0.07, 0.32)	.220
HADS-D				
+ age, sex	0.20 (0.01, 0.39)	.140	−0.12 (− 0.31, 0.07)	.396
+ multiv. Adj.	0.17 (− 0.03, 0.37)	.151	− 0.16 (− 0.34, 0.03)	.151

*WEMWBS* Warwick–Edinburgh Mental Wellbeing Scale, *HADS-A/D* Hospital Anxiety and Depression Scale-Anxiety/Depression subscale, *CI* confidence interval. *multiv. adj* multivariate adjustment for age, sex, BMI, limiting illness or disability, and educational attainment

Estimates in bold type are statistically significant. *P*-values are corrected for multiple comparisons

In the LBC1936, wellbeing score, which was assessed concurrently with sedentary behaviour, was not associated with sedentary time or number of sit-to-stand transitions.

HADS anxiety score (assessed concurrently with sedentary behaviour) was not associated with sedentary time in the LBC1936 cohort. Similarly, in the Twenty-07 1930s and 1950s cohorts, HADS anxiety score (assessed 8 years previously) was not associated with sedentary time. HADS anxiety score was also not associated with number of sit-to-stand transitions in any of the three cohorts.

HADS depression score (assessed concurrently with sedentary behaviour) was positively associated with sedentary time following adjustment for age and sex in the LBC1936 cohort (*β* = 0.14, *p* = 0.045). HADS depression score (assessed 8 years previously) was also associated with sedentary time, following adjustment for age and sex, in the Twenty-07 1950s (*β* = 0.19 (*p* = 0.004) cohort. These associations were non-significant in all three cohorts following additional adjustment for BMI, education, and limiting illness or disability. HADS depression score (assessed 8 years previously) was not associated with number of sit-to-stand transitions in the Twenty-07 cohorts; however, we did observe a significant inverse association between HADS depression score (assessed concurrently with sedentary behaviour) and number of sit-to-stand transitions in the LBC1936 cohort. This association was significant in the age- and sex-adjusted model (*β* = − 0.17, *p* = 0.024), and the fully adjusted model (*β* = −0.19, *p* = 0.012).

### Sensitivity analysis

Associations between HADS depression score and sedentary outcomes could be driven by the “I feel as I am slowed down” item of the HADS depression scale. To test for this effect, models of the associations between depression and sedentary outcomes were repeated replacing the HADS depression scale with a modified version that excluded the item related to sedentary behaviour. *P*-values were corrected for multiple comparisons [60]. In the LBC1936, the association between HADS depression score and sedentary time was no longer significant in the age- and sex-adjusted model (*p* = 0.066). The association between HADS depression score and number of sit-to-stand transitions was non-significant in the age and sex adjusted model but remained significant in the fully adjusted model (*p* = 0.030). In the Twenty-07 1950s cohort, the association between HADS depression score and sedentary time was slightly attenuated but remained significant (*β* =  0.17, *p* = 0.016). In the Twenty-07 1930s cohort, associations between HADS depression score and sedentary time or number of sit-to-stand transitions remained non-significant.

### Meta-analysis

**Fig. 1:**
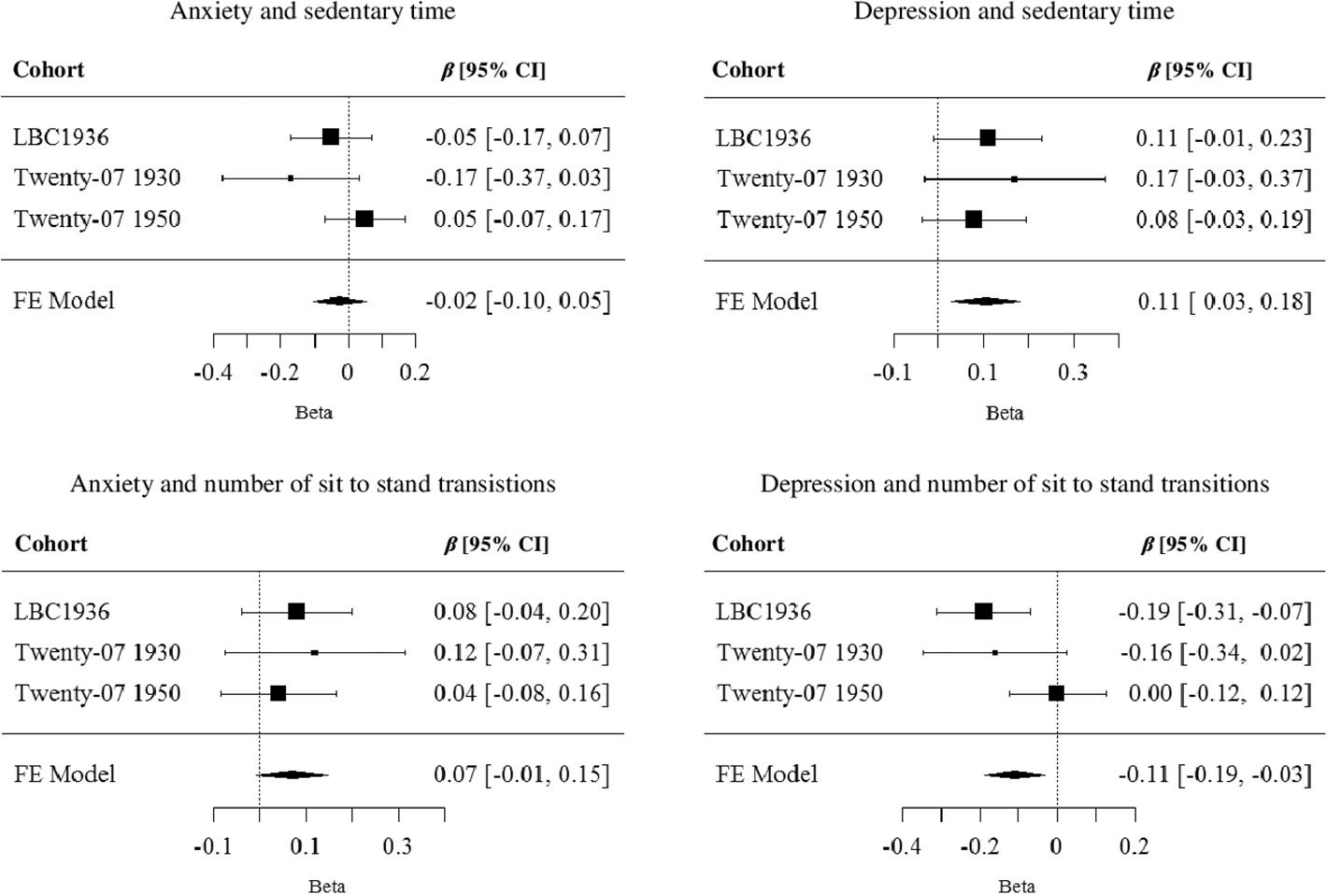
Forest plots of the association between anxiety and sedentary time, anxiety and number of sit-to-stand transitions, depression and sedentary time, and depression and number of sit-to-stand transitions

We meta-analysed estimates of the associations between HADS subscales and sedentary time and sit-to-stand transitions. See Fig. 1 for forest plots of our results. Estimates were adjusted for age, sex, BMI, education, and limiting illness or disability. HADS anxiety score was not significantly related to sedentary time or number of sit-to-stand transitions. The meta-analytic estimates for these associations were *β* = − 0.02 (95% CI = − 0.10, 0.05) and *β* = 0.07 (95% CI = − 0.01, 0.15) respectively. Depression score was positively associated with sedentary time and negatively associated with number of sit-to-stand transitions, with meta-analytic estimates of *β* = 0.11 (95% CI = 0.03, 0.18) and *β* = − 0.11 (95% CI = − 0.19, −0.03) respectively. These estimates were only slightly weaker when we meta-analysed results from analysis with the modified depression score, excluding the “I feel as I am slowed down” item. Meta-analytic estimates from this analysis were *β* = 0.10 (95% CI = 0.02, 0.17) for sedentary time and *β* = − 0.09 (95% CI = − 0.17, − 0.01) for number of sit-to-stand transitions.

## Discussion

The aim of this study was to test for an association between wellbeing or symptoms of anxiety and depression and objectively measured sedentary behaviour or sit-to-stand transitions in three cohorts of older adults. No evidence of an association between wellbeing or symptoms of anxiety and sedentary behaviour or sit-to-stand transitions was found. However, participants who reported higher depressive symptoms were more likely to be sedentary and make fewer sit-to-stand transitions.

The finding that wellbeing was not related to sedentary time or number of sit-to-stand transitions measured concurrently is in line with reports from two previous studies of older adults [37, 38]. Both studies found no association between life satisfaction or wellbeing and between participant differences in sedentary behaviour. Withall et al. [37] conclude that although higher wellbeing is linked to greater engagement in leisure and social activities in older age [61], this association may not translate into reduced sedentary behaviour because physical activity is not a popular leisure pursuit among older adults [62]. By contrast, one study did find a negative association between a momentary measure of positive affect and sedentary behaviour in a sample of women aged between 40 and 60 [36]. It is possible that the effect reported by this latter study is specific to middle aged women. Alternatively, sedentary behaviour may be more closely related to momentary measures of positive affect than retrospective measures of wellbeing such as the WEMWBS – which requires participants to recall how they have felt over the past 2 weeks.

Symptoms of anxiety were not associated with sedentary time or number of sit-to-stand transitions in any of the three cohorts. These associations were also non-significant in the meta-analysis of estimates across cohorts. Some cross-sectional studies found a positive association between symptoms of anxiety and sedentary behaviour [31]. However, none of these studies tested for an association between anxiety and sedentary behaviour in older adults. The null finding in the present study could indicate that this association is specific to younger populations. Another study with Seniors USP participants, found that fear of crime was associated with more sedentary behaviour among retired Twenty-07 1950s cohort members [63]. It is possible that the association between anxiety and sedentary behaviour is dependent on the cause of anxiety. Anxiety specifically related to one’s neighbourhood may be more closely related to sedentary behaviour than other forms of anxiety.

Regardless of age and sex, LBC1936 and Twenty-07 1950s participants with more depressive symptoms were more sedentary. LBC1936 participants with more depressive symptoms also made fewer sit-to-stand transitions. This association was not fully explained by differences in age, sex, BMI, history of limiting long-standing illness or educational attainment. Meta-analytic estimates of the association between depressive symptoms and sedentary time or number of sit-to-stand transitions were significant – suggesting that depressive symptoms are related to sedentary behaviours and that these associations are partially independent of age, sex, BMI, long standing illness, and educational attainment. Previous findings indicate that, among older adults, depressive symptoms are associated with some self-reported sedentary activities such as watching television [29, 32, 33]. The findings of the present study demonstrate that depressive symptoms are also positively associated with objectively measured sedentary time and sit-to-stand transitions. In contrast with this result, Rosenberg et al. [34] found no association between an objective measure of sedentary behaviour and depressive symptoms. This latter study may have under-estimated the association between depressive symptoms and sedentary behaviour as the sedentary behaviour measure (hip-worn accelerometer) was not sensitive to changes in posture.

Because sedentary behaviour was only assessed at one time point, it was not possible to examine the direction of its association with depression. Sedentary behaviour could be reciprocally related to symptoms of depression. Specifically, the experience of depressive symptoms such as negative affect, apathy, and low energy could cause an individual to disengage from physical activity and become more sedentary [26, 64, 65]. In addition, frequent engagement in some types of sedentary behaviour such as watching television could increase social isolation, which, in turn, could increase the risk of depression [66]. A further possibility is that sedentary behaviour and depressive symptoms are not causally related, but are both impacted by other factors. In our study, we controlled for the potentially confounding effect of age, sex, BMI, long standing illness, and educational attainment. However, other factors, previously associated with sedentary behaviour and risk of depression, such as sleep duration or fear of crime could also play a role [63, 67–71].

The present study had several strengths. Firstly, the data included three distinct psychosocial constructs: wellbeing, and symptoms of anxiety and depression. In addition, we were able to test whether prior or concurrent measures of anxiety or depression are related to sedentary behaviour. Finally in contrast with most previous studies in this area, sedentary behaviour was assessed objectively rather than by self-report. A number of limitations should also be noted. Firstly, although postural changes were assessed objectively with activPAL activity monitors, waking time was self-reported, this may have reduced the reliability of the sedentary behaviour measure. In addition, type of sedentary behaviour was not recorded. It is possible that the psychosocial factors in the present study were related to some types of sedentary behaviour (e.g., watching television) but not others (e.g., socialising). Furthermore, it should be noted that the sit-to-stand transitions measure did not distinguish between participants who spent long uninterrupted periods of time standing, and those who spent long uninterrupted periods of time sitting: both groups would be classified as making few sit-to-stand transitions. Thus, this measure should be considered as distinct from measures of actual time spent sedentary. Finally, in the case of Twenty-07 cohorts, symptoms of depression and anxiety were recorded 8 years prior to the assessment of sedentary time and sit-to-stand transitions. Therefore, a key assumption of our study, was that participants’ depressive and anxiety symptoms would remain stable over the 8 year follow-up period. In support of this assumption, previous work has documented the stability of these constructs in samples of older people over extended follow-up periods [72–74]. Effect sizes for the association between depressive symptoms and sedentary time or sit-to-stand-transitions were similar in the LBC1936 cohort (where symptoms of depression were assessed concurrently with sedentary behaviour) and the Twenty-07 1930 cohort, but weaker in the Twenty-07 1950 cohort. It is possible that we would have detected stronger associations in the Twenty-07 cohorts had symptoms of depression been assessed at the same time as the sedentary behaviour outcomes.

## Conclusion

Reducing sedentary behaviour among older adults is an important public health objective. Although further research is needed to establish whether depression and sedentary behaviour are causally related, the findings of this study indicate that depressive symptoms could play a role in determining sedentary patterns in later life.

## Abbreviations

BMI: Body mass index
FDR: False discovery rate
HADS: Hospital anxiety and depression scale
Twenty-07 1950s: West of Scotland twenty-07 1930s cohort
Twenty-07 1950s: West of Scotland twenty-07 1950s cohort
USP: Understanding sedentary patterns
WEMWBS: Warwick–Edinburgh mental wellbeing scale
